# Transperitoneal Approach versus Retroperitoneal Approach: A Meta-Analysis of Laparoscopic Partial Nephrectomy for Renal Cell Carcinoma

**DOI:** 10.1371/journal.pone.0091978

**Published:** 2014-03-21

**Authors:** Tong Ren, Yan Liu, Xiaowen Zhao, Shaobin Ni, Cheng Zhang, Changgang Guo, Minghua Ren

**Affiliations:** 1 Department of Urinary Surgery, First Affiliated Hospital, Harbin Medical University, NanGang District, Harbin, Heilongjiang Province China; 2 Department of Biostatistics, School of Public Health, Harbin Medical University, Nangang District, Harbin, China; 3 Department of Health Economics, School of Public Health, Harbin Medical University, Nangang District, Harbin, China; University of British Columbia, Canada

## Abstract

**Objective:**

To compare the efficiency and safety of the transperitoneal approaches with retroperitoneal approaches in laparoscopic partial nephrectomy for renal cell carcinoma and provide evidence-based medicine support for clinical treatment.

**Methods:**

A systematic computer search of PUBMED, EMBASE, and the Cochrane Library was executed to identify retrospective observational and prospective randomized controlled trials studies that compared the outcomes of the two approaches in laparoscopic partial nephrectomy. Two reviewers independently screened, extracted, and evaluated the included studies and executed statistical analysis by using software STATA 12.0. Outcomes of interest included perioperative and postoperative variables, surgical complications and oncological variables.

**Results:**

There were 8 studies assessed transperitoneal laparoscopic partial nephrectomy (TLPN) versus retroperitoneal laparoscopic partial nephrectomy (RLPN) were included. RLPN had a shorter operating time (SMD = 1.001,95%confidence interval[CI] 0.609–1.393,P<0.001), a lower estimated blood loss (SMD = 0.403,95%CI 0.015–0.791,P = 0.042) and a shorter length of hospital stay (WMD = 0.936 DAYS,95%CI 0.609–1.263,P<0.001) than TLPN. There were no significant differences between the transperitoneal and retroperitoneal approaches in other outcomes of interest.

**Conclusions:**

This meta-analysis indicates that, in appropriately selected patients, especially patients with intraperitoneal procedures history or posteriorly located renal tumors, the RLPN can shorten the operation time, reduce the estimated blood loss and shorten the length of hospital stay. RLPN may be equally safe and be faster compared with the TLPN.

## Introduction

Renal cell carcinoma (RCC) was thought to arise primarily from the proximal convoluted tubules and some histologic subtypes were from the more distal components of the nephron. RCC, which accounts for 2%–3% of all adult malignancies, with the highest incidence occurring in Western countries [1], is the most lethal of the urologic cancers. Traditionally, more than 40% of patients with RCC have died from their cancer, in contrast with the approximately 20% mortality rates associated with prostate and bladder carcinomas [2]. The incidence rates of RCC have risen steadily during the last three decades in most of the world, with a mean increase of 2–3% per year [3]. Surgical removal of the kidney (radical nephrectomy) or the tumor (partial nephrectomy, PN) is the only curative therapeutic approach for RCC [1]. In 1991, Clayman et al. [4] executed the first transperitoneal laparoscopic nephrectomy. Since then, laparoscopic nephrectomy has gained increasing worldwide acceptance because of its benefits in terms of patient recovery and perioperative morbidity [4], [5]. Long-term oncological studies have shown that the outcomes of laparoscopic nephrectomy are similar to those of open surgery [6]–[11]. Therefore, laparoscopic nephrectomy has displaced the open surgery and became the basic operation for RCC. Due to the increased detection of tumors by imaging techniques, such as ultrasound and computed tomography, the number of incidentally diagnosed RCCs has increased, largely owing to a more prevalent use them for the evaluation of a variety of abdominal or gastrointestinal complaints [12]. These tumors are more often small, slow growth and of lower stage such as T1 [13].The nephron sparing surgery (NSS) can resects the localized tumors completely and retain the functional renal sections. NSS emerged as a new surgical approach for T1 RCC and became a basic treatment [12]. The laparoscopic partial nephrectomy can be executed from transperitoneal and retroperitoneal approach. The first transperitoneal laparoscopic partial nephrectomy (TLPN) was reported by Winfield et al. 1993 [14]. The retroperitoneal laparoscopic partial nephrectomy (RLPN) was first reported by Gill et al. in 1994 [15]. Although laparoscopic partial nephrectomy appeared earlier, but its development is relatively slow, this lag is mainly technical reasons. In recent years, with the rapid development of endoscopic equipment and the progress of surgeon surgical techniques, TLPN and RLPN have become the mature technology and main treatment for T1 RCC. We collected related literatures and made a meta-analysis to compare the efficiency and safety of the transperitoneal approaches with retroperitoneal approaches in laparoscopic partial nephrectomy for renal cell carcinoma.

## Materials and Methods

### Literature Search

A systematic computer literature search of EMBASE, MEDLINE and Cochrane library was executed to identify relevant studies. No time or language restrictions were applied. The search terms were ‘(transperitoneal OR retroperitoneal) [Title/Abstract] AND (laparoscopic partial nephrectomy) [Title/Abstract] AND (nephron sparing surgery OR heminephrectomy) [Title/Abstract].’ Articles were also identified using the ‘related articles’ function.

### Inclusion Criteria

There were four inclusion criteria used:(1)the literatures compared RLPN with TLPN,(2) evaluation of at least one of the outcomes of interest mentioned below,(3) a randomized controlled trial or retrospective comparative study design,(4) patients must had preoperative staging including CT or MRI according to the TNM classification, and had a clinical stage of T1.

### Exclusion Criteria

There were four exclusion criteria were used: (1)incomplete documentation and contact the authors did not reply,(2) When two studies were reported by the same institution and/or authors, the most recent report was used. (3) Laparoscopic partial nephrectomy for benign lesions, (4) The inclusion criteria were not met.

### Data Extraction

Two reviewers independently screened, evaluated the included studies and extracted data from them, and disagreements were resolved by discussion until a consensus was reached. The following information was extracted from each study: first author; year of publication; inclusion and exclusion criteria; matching criteria; study design; characteristics of the study population; number of patients in each group; and outcomes of interest. When the data of the literature was incomplete, we contacted the corresponding authors, but no one provided any additional information.

### Outcomes

The following outcomes were used to compare RLPN and TLPN. (1) Perioperative variables: operating time, estimated blood loss (EBL) and warm ischaemia time (WIT),(2) Postoperative variables: length of hospital stay (LOS) and postoperative serum creatinine (SCr) level,(3) Surgical complications: overall complication rate, open conversion rate and blood transfusion rate,(4) Oncological variables: overall recurrence rate and positive margin rate.

### Statistical Analysis

This meta-analysis was executed according to the recommendations of the Cochrane Collaboration and the Quality of Reporting of Meta-analyses (QUORUM) guidelines [16], [17]. The odds ratios (ORs) and weighted mean differences (WMDs) and the were used to compare dichotomous and continuous variables, respectively. The standardized mean differences (SMDs) were used when continuous variables were measured in different units, the outcomes was reported with 95% CIs. The statistical heterogeneity between all studies was evaluated by using the chi-squared test with significance set at P<0.10, and the quantity of heterogeneity was evaluated using the I^2^ statistic. We reported random-effects model (RE) if there was heterogeneity between studies(I^2^>50). Otherwise, we reported the fixed-effects model (FE) [18]. The quality of studies was assessed by examining three aspects: patient selection, comparability of the study groups, and evluation of outcomes. The score of each study was allocated from 0 to 9 (Table 1). Statistical analysis was executed using the procedure STATA 12.0.

**Table 1:** Characteristics of included studies.

Studies [reference]	year	Study type	No. patients TLPN/RLPN	Clinical stage	Comparability	Study quality	Variables	Tumor mean size(cm) TLPN/RLPN
Jonathan Wright [19]	2005	Retrospective	19/32	T1a	1,2,4,5,6,7	6	1,2,3,4,6,7	2.67/2.09
Christopher NG [20]	2005	Retrospective	100/63	T1a	1,2,3,4,5,6,7,9	8	1,2,3,4,5,6,7	3.10±1.00/2.60±0.90
Kathleen Kieran [21]	2007	Retrospective	45/27	T1a	1,2,4,6,7	6	1,2,3,4,5,6,7	2.66±1.20/2.05±0.84
Martin Marszalek [22]	2010	Retrospective	35/70	T1a	1,2,3,4,5,6,7	7	1,2,3,4,6,7	2.40/2.50
Emara AM [23]	2011	Retrospective	6/27	T1a	NA	4	2,6,	2.65/2.60
Tugcu V [24]	2011	Retrospective	26/23	T1a	NA	4	1,2,3,4,6,	2.88/2.47
Idir Ouzaid [12]	2012	Retrospective	66/87	T1a	1,2,3,4,5,6,7,9	7	1,2,3,4,6,	2.64±1.07/2.70±1.25
Archie Hughes-Hallett [25]	2013	Retrospective	59/44	T1a	1,2,4,5,7	5	1,2,3,4,6,	3.07/2.84

The score of each study was allocated from 0 to 9 according on the modified Newcastle–Ottawa Scale and showed in Study quality.

Comparability:1 = age, 2 = gender, 3 = BMI, 4 = clinical stage, 5 = tumor side, 6 = tumor position, 7 = tumor size, 8 = previous abdominal surgery history, 9 = ASA score, NA = data not available.

Variables:1 = operating time, 2 = warm ischemic time, 3 = estimated blood loss, 4 = hospital stay, 5 = postoperative SCr level, 6 = complications, 7 = Positive margin.

Study quality: The score of each study was allocated from 0 to 9 according on the modified Newcastle-Ottawa scale.

## Results

**Figure 1:**
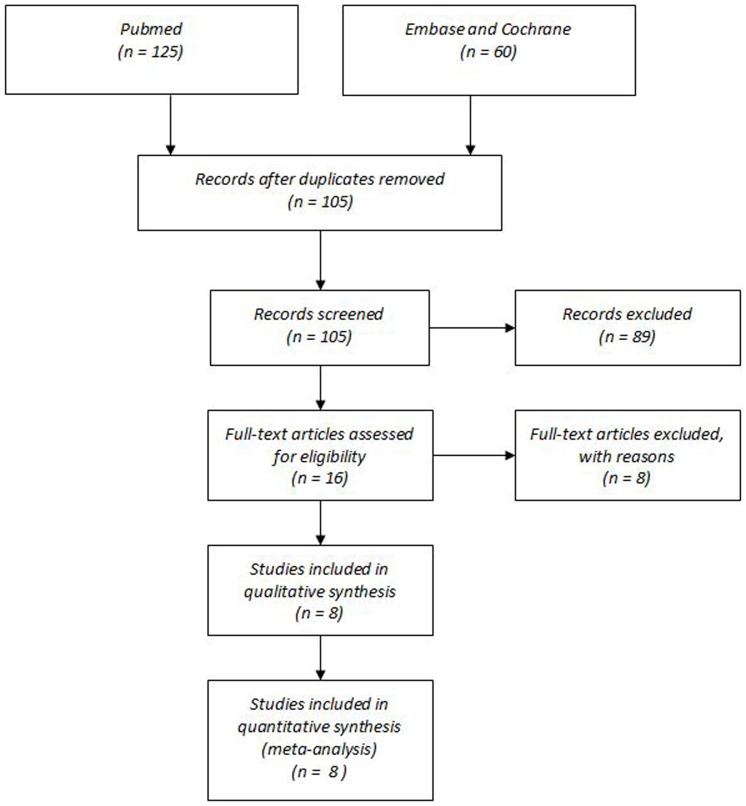
Flowchart of selection of studies for inclusion in meta-analysis.

A total of 185 literature published from 2004 to 2013 were searched, there were 8 studies fulfilled the inclusion criteria after screened and were included in this meta-analysis (Fig. 1). Examination of the reference lists of these studies did not detect any further studies for evaluation. There were 706 patients undergone partial nephrectomy, 356 patients were TLPN and 350 patients were RLPN. The characteristics of included literatures are shown in table 1. All the studies were retrospective observational studies. For the retrospective observational studies, the risk of bias was evaluated using the modified Newcastle–Ottawa Scale. None of these studies adopted an appropriate protocol for treatment assignment, with allocation being at the physician's discretion in studies.

### Meta-Analysis of Perioperative Variables

**Figure 2:**
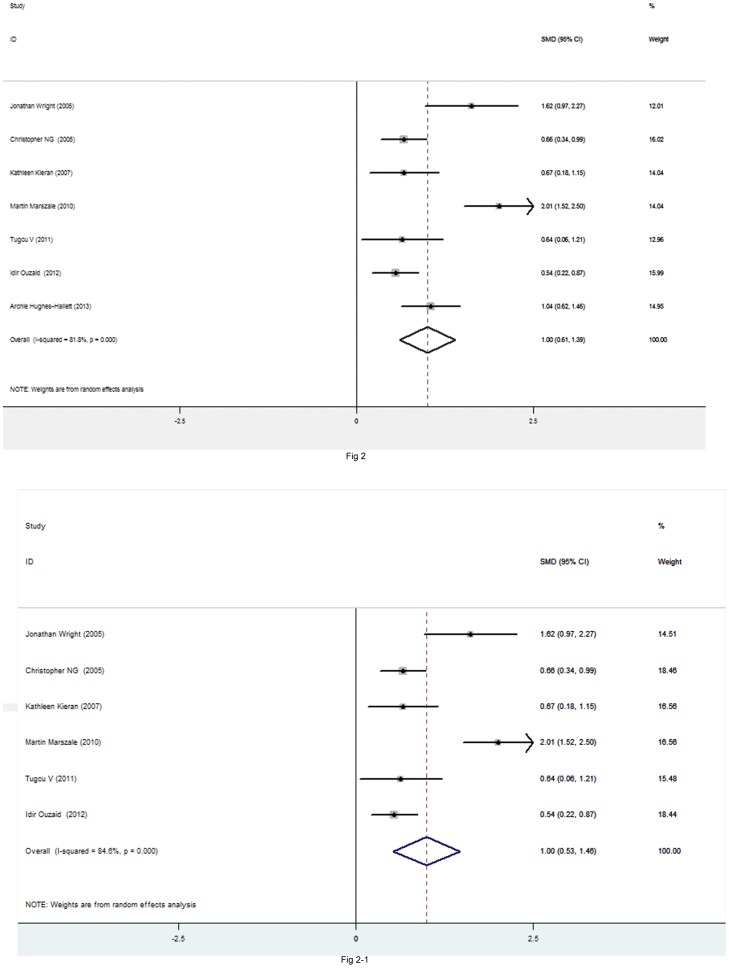
Forest plots of operating time TLPN vs RLPN using a random-effect model. Squares indicate study-specific risk estimates (size of the square reflects the study-specific statistical weight, i.e., the inverse of the variance); horizontal lines indicate 95% confidence intervals (CIs); diamonds indicate summary risk estimate with its corresponding 95% confidence interval.

Pooled data from the seven studies [12], [19]–[22], [24]–[25] that reported operating time for PN showed that operating time was significantly shorter in RLPN than TLPN (SMD = 1.001, 95%CI 0.609–1.393, P<0.001). When we exclude one study [25] which used the robotic approach, the result in statistical analysis is not changed (SMD = 0.99, 95%CI 0.533–1.465, P<0.001). Fig. 2 and Fig. 2-1.

**Figure 3:**
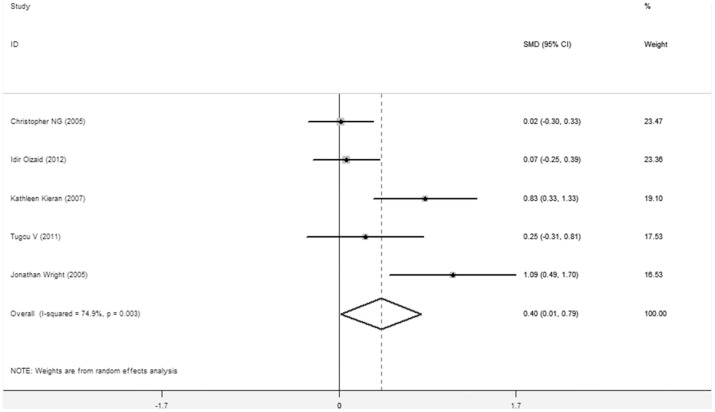
Forest plots of estimated blood loss TLPN vs RLPN using a random-effect model. Squares indicate study-specific risk estimates (size of the square reflects the study-specific statistical weight, i.e., the inverse of the variance); horizontal lines indicate 95% confidence intervals (CIs); diamonds indicate summary risk estimate with its corresponding 95% confidence interval.

Pooled data from the five studies [19]–[21], [24]–[25] that reported EBL for PN showed a significant difference between TLPN and RLPN (SMD = 0.403, 95%CI 0.015–0.791, P = 0.042). One study [25] shows the result as mean EBL (ml):TLPN 395.1(20–3100) vs. RLPN 88.0(20–1600). One study [22] shows the result as mean decline in % of baseline hemoglobin: TLPN 17.1% vs. RLPN 16.6%. Fig. 3

Pooled data from the six studies [12], [19]–[21], [23]–[24] that reported WIT for PN showed no significant difference between TLPN and RLPN (SMD = 0.302, 95%CI −0.340–0.945, P = 0.356). One study [25] shows the result as mean WIT (min): TLPN 19.1(0–40) vs. RLPN 22.1(0–48).

### Meta-Analysis of Postoperative Variables

**Figure 4:**
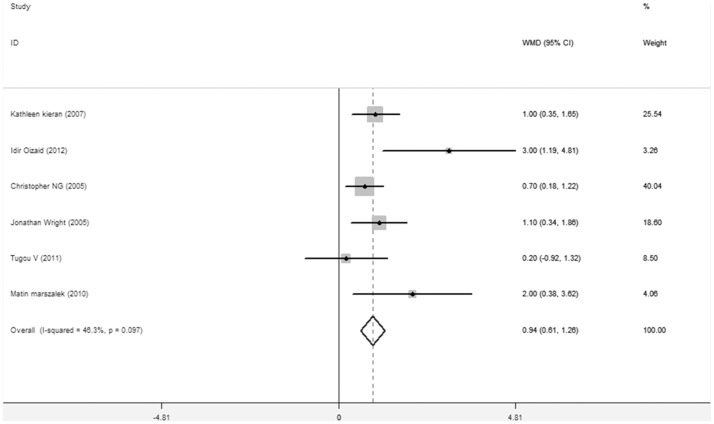
Forest plots of length of hospital stay TLPN vs RLPN using a fixed-effect model. Squares indicate study-specific risk estimates (size of the square reflects the study-specific statistical weight, i.e., the inverse of the variance); horizontal lines indicate 95% confidence intervals (CIs); diamonds indicate summary risk estimate with its corresponding 95% confidence interval.

Six studies [12], [19]–[21], [24] reported the LOS. There was a significant difference between TLPN and RLPN in LOS (WMD = 0.936 DAYS,95%CI 0.609–1.263,P<0.001). One study [25] shows the result as mean LOS (days): TLPN 4.6(1–28) vs. RLPN 2.5(1–50). Fig. 4.

Pooled data from the two [20], [21] studies that reported postoperative SCr level for PN showed no significant difference between TLPN and RLRN (WMD 0.02 mg/dL; 95% CI −0.08–0.11;P = 0.68).

There were no significant differences between TLPN and RLPN in overall complication rate, postoperative complication rate, or open conversion rate (Table 2).

**Table 2:** Overall analysis of TLPN vs. RLPN.

Outcome	No. of studies	TLPN/RLPN	Statistical results			Study heterogeneity			
			Statistic	Value(95%CI)	*P*	*χ* ^2^	*df*	*I* ^2^(%)	*P*
Operating time (min)	8	356/350	SMD	1.001(0.609,1.393)	P<0.001	33.5	6	81.8	<0.001
WIT(min)	7	349/323	SMD	0.302(−0.340,0.945)	P = 0.356	93.89	6	93.6%	<0.001
EBL(ml)	5	262/236	SMD	0.403(0.015,0.791)	P = 0.042	15.94	4	74.9%	0.003
LOS(day)	6	291/302	WMD	0.936(0.609,1.263)	P<0.001	9.31	5	46.3%	0.097
PostoperativSCr(mg/dl)	2	145/90	WMD	0.02 (−0.08,0.11)	P = 0.68	1.16	1	14%	0.28
Overall complications	6	324/323	OR	0.849(0.576,1.250)	P = 0.406	1.94	5	0.0%	0.857
Intraoperative complications	4	170/149	OR	2.30 (0.83,6.4)	P = 0.11	3.58	3	16%	0.31
Postoperative complications	4	199/192	OR	1.33 (0.73,2.41)	P = 0.35	3.09	3	3%	0.38
Conversion rate	5	205/219	OR	2.14 (0.85,5.39)	P = 0.11	2.93	4	0%	0.57
Positive margin rate	4	199/192	OR	1.29 (0.48,3.46)	P = 0.61	1.22	3	0%	0.75

Pooled data from the four studies [19]–[22] that reported positive surgical margin rates for PN showed no significant difference between TLPN and RLPN (FE: OR = 1.29; 95% CI 0.48–3.46;P = 0.03). None of the six studies assessing PN reported recurrence or survival rates, making it impossible to perform meta-analysis on these outcomes.

### Sensitivity Analysis and Publication Bias

**Figure 5:**
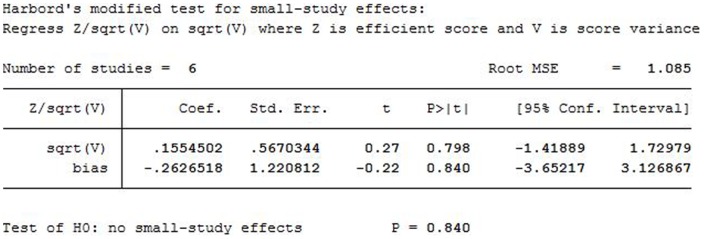
The result of publication bias in hardord way.

Only three studies assessing PN scored ≥7 stars on the modified Newcastle–Ottawa Scale (Table 1), so it was not appropriate to perform sensitivity analysis on this group. The result of publication bias checkout in hardord way for overall complication rate is P = 0.840, the study outcomes were within the 95% CIs and were distributed symmetrically, showing no evidence of publication bias. Fig. 5.

## Discussion

This study is not without its limitations. The following limitations that must be taken into account. First, this is a retrospective study and all the literatures were observational. The lack of randomized controlled studies which are high quality medicine evidence made the quality of our study and data is not very high, and the absence of allocation concealment and blinding may have influenced the measurement of postoperative variables. Studies were performed with varying protocols and different level of surgical expertise. Second, computer-based literature searching is performed, but we can not guarantee that all the relevant literatures have been searched and included in this study. Third, heterogeneity between studies was marked for all the continuous variables. There was significant variability in terms of definitions, inclusion criteria, exclusion criteria, operating technique, and measurement of outcomes. It was impossible to match all patient groups for age, BMI, and previous abdominal history. All these factors could have contributed to the high heterogeneity between studies. Use of the random model for pooled data might minimize the effects of heterogeneity, but can not eliminate them. The degree of heterogeneity fell for most outcomes with sensitivity analysis, but this difference was not significant. Fourth, some data were reported as median (range), which may be because these variables were not normally distributed. We calculated the mean (SD) values from data ranges, or P values, therefore, the bias of the pooled effect should be considered. Finally, some authors did not report the proportion of patients lost to follow-up, which may influence the reliability of the conclusions.

Endoscopic PN can be performed via the transperitoneal or retroperitoneal approach, each providing specific advantages and disadvantages.

The goal of laparoscopic partial nephrectomy should be safe removal of the renal segment in question, while maintaining acceptable hemostasis, adequate operative visualization and closure of any entry into the collecting system. Arguments in favor of the transperitoneal route are the lager working space, allowing for wider angulation and maneuverability with laparoscopic instruments, and the more accustomed orientation by familiar anatomic landmarks, [19], [21] but requires bowel mobilization to expose the kidney. Intra-abdominal adhesions, which might develop as result of laparoscopic surgical procedures, usually appear to be of minor clinical significance [26]. Retroperitoneoscopy, by avoiding bowel mobilization, seems to provide a more direct access to the kidney and the renal hilum [19]. Drawbacks are the spatial limitations of the narrow retroperitoneal working space, [27] the lack of view, and the risk of disorientation and causing inadvertent injury. Select tumors may be approached by either route, according to the surgeons' preference. However, similar to open surgery, indication and patient selection for either access have to be carefully considered by the endoscopic surgeon, with tumor localization as most important decisive factor, is recognized as shortcoming of the present retroperitoneal analysis. The choice of the laparoscopic approach was at the discretion of the surgeon, and it was dictated primarily by the location and technical complexity of the renal mass. The transperitoneal approach was generally used for anterior or lateral lesions. The retroperitoneal approach was generally used for posterior, posteromedial, or posterolateral lesions. Furthermore, transperitoneal and retroperitoneal PN were performed by experienced laparoscopic surgeons, but at different institutions, a limitation of the present study that has to be considered when interpreting the data provided. However, patients in studies which we included were not pair matched well, many factors can affect the outcomes such as the tumor size. But the data about tumor size in all included literatures were incomplete; we got the mean size without the standard deviation only, so we could not perform an accurate statistical analysis. For careful consideration, we compared the tumor mean size, TLPN and RLPN group value is 2.84 cm and 2.54 cm respectively, and the transperitoneally accessed tumors were a little larger. From urology professional point of view, and based on the experience of our operation, if the difference of mean size is 0.3 cm for tumor which is about 3.0 cm, the outcomes such as operating time, WIT, EBL etc will not be influenced significantly. Variations in surgical outcomes between these groups likely reflect the characteristics of the treated lesion as well as of the surgical procedure. This meta-analysis included 8 studies comparing RLPN with TLPN. The results showed that RLPN had a shorter operating time, a lower EBL and a shorter LOS than TLPN. We found no significant differences between the groups in other outcomes.

According to our initial analysis, RLPN had a shorter operating time than TLPN, and the difference in operating time was probably clinically significant. As metioned before, anteriorly located tumors, deeply infiltrating tumors are typically approached transperitoneally, and posterior is likely to be treated with RLPN. All of the studies included in the meta-analysis evaluated patients with T1a tumors only. In the included studies, the retroperitoneal approach was selected primarily for posteriorly located tumors. The tumors which treated with TLPN may anteriorly located or deeply infiltrated could extend the operating time. RLPN is more difficult than TLPN for many surgeons because of its lots of limitations. The skills necessary for TLPN are rapidly acquired, and decreasing operating time with the RLPN suggests a learning curve for this procedure as well. The data and recently published work [20] suggest that experienced surgeons can overcome these limitations to achieve statistically equivalent outcomes with RLPN and TLPN [21]. The RLPN route can provide a more direct access to the kidney and avoid bowel mobilization to decrease the operating time. It is very difficult for the patients with past history of intraperitoneal procedures to perform TLPN. An RP nephrectomy may be especially useful in patients with prior abdominal surgery or radiation, as convalescence and recovery of bowel function are not prolonged with this approach [21]. The cautious conclusion is that RLPN is faster than TLPN for special patients.

Two important factors for the Endoscopic PN are low EBL and short kidney warm ischemia time. The EBL was significantly lower in RLPN than TLPN. The reduction in EBL in the retroperitoneal group was thought to be related to the reduction in surgical dissection and may also have been a refection of occasional, but clearly important, EBL associated with the early unclamping technique used by the surgeons performing a solely transperitoneal approach. The LOS was also significantly shorter in RLPN than TLPN. The RLPN can reduce the damage of gastrointestinal function by avoiding bowel mobilization and make the patients exhaust and defecate earlier, thus feeding earlier and recover more quickly. Shekarriz et al [28] showed 10–40 minutes kidney warm ischemia time has no impact on renal function change. There were no significant differences between RLRN with TLPN in WIT, postoperative SCr level.

The most common complication following open partial nephrectomy is urine leakage with a mean reported incidence of 6.5%(range1.4% to 17%) [29]. In the laparoscopic literature the urine leakage rate in early series was 5.9% to 28.5% [19]. However, in recent series that have used collecting system overawing the urine leakage rate is 0% to 2% [19]. The overall and intraoperative complication rates were not significantly different between RLPN and TLPN. Unfortunately, most of the studies reported overall complication rate without reporting the specific events, which may have introduced bias, but our analysis indicates that RLRN is at least as safe as TRLN. The negative surgical margin after LPN is the key for RCC treatment. Compared with OPN, LPN for T1a tumors had the same oncologic results. Our study showed no significant difference between TLPN and RLPN. In previously published comparative series, the RLPN and TLPN were similar in terms of oncological outcomes. Our analysis confirmed these results. As tumor location and the size were the primary factors surgeons used in selecting the approach, the TLPN used more often in anterior, lateral or complicated cases, the distribution of tumor location in the two groups was quite different. As mentioned before, tumors in their analysis were a little lager in the transperitoneal group, which might have contributed to these results. This might have been a source of bias, and the meaningful results of the comparison of RP and TP approach such as operating time, EBL, LOS can be affected. However, the most direct route to the renal mass should facilitate dissection and minimize manipulation, thereby limiting complications, so we believe that our comparison of RP and TP patients is appropriate despite this limitation.

In the United States and other developed countries, LPN is being widely replaced by robotic partial nephrectomy, but restricted by economic level and medical technology; LPN is the primary surgical method in China and other developing countries. But the robotic partial nephrectomy will hit the mainstream because its advantage of the inherent. The disadvantage of RLPN is small working place which become an outstanding feature of robotic approach. The robotic partial nephrectomy combines all these merits but does not have the defects versus the pure LPN such as: the more accurate and flexible operation, the less trauma, surgical indications wider, the less body damage and so on. It is not difficult at all to foresee that a wide range of popularization and development of the robotic partial nephrectomies will show.

## Conclusion

LPN has become the recommended treatment for amenable T1 RCC. According to our analysis, RLPN has a shorter operating time, lower EBL and shorter LOS than TLPN. As tumor location is the primary factor surgeons used in selecting the approach. For the patients with intraperitoneal procedures history or posteriorly located tumors, the RLPN may be faster and equally safe compared with the TLPN and can make patients recover more quickly. As the inherent limitations, the conclusions drawn from our pooled results should be cautious and need higher quality studies to be further confirmed.

## Supporting Information

Checklist S1 **PRISMA checklist.**
(DOC)
